# An adapted version of the long International Physical Activity Questionnaire (IPAQ-L): construct validity in a low-income, multiethnic population study from Oslo, Norway

**DOI:** 10.1186/1479-5868-4-13

**Published:** 2007-04-20

**Authors:** Sidsel Graff-Iversen, Sigmund A Anderssen, Ingar M Holme, Anne Karen Jenum, Truls Raastad

**Affiliations:** 1 Division of Epidemiology, Norwegian Institute of Public Health, PO Box 4404 Nydalen, 0403 Oslo, Norway; 2 Department of Sports Medicine, Norwegian School of Sports Sciences, PO Box 4014, Ullevål Stadion, 0807 Oslo, Norway; 3 Diabetes Research Centre, Aker and Ullevål University Hospital, University of Oslo, Norway; 4 Department of Physical Performance, Norwegian School of Sports Sciences, PO Box 4014, Ullevål Stadion, 0807 Oslo, Norway

## Abstract

**Background:**

The aim was to assess the construct validity characteristics of an adapted version of the long International Physical Activity Questionnaire (IPAQ-L) and report seasonal variations in physical activity (PA).

**Methods:**

In two multiethnic suburbs of Oslo, Norway, all men and women aged 31–67 years (N = 6140) were invited to a survey in 2000, and participants (N = 2950) were re-invited in 2003. Complete IPAQ-L forms were delivered by 2274 baseline participants. We used the first IPAQ-L version, which asks for PA in a usual week with separate answering alternatives for summer and winter. Baseline energy expenditure calculated from IPAQ-L was compared with anthropometrical and biological measurements including maximal aerobic power in a subgroup, and individual changes in PA were compared with changes in these measurements.

**Results:**

Vigorous PA within all domains, leisure-time PA (LPA), total PA, and in men occupational PA correlated with waist-to-hip ratio (rho around -0.1, p < 0.05). For vigorous PA and LPA similar correlations were found with triglycerides and high-density lipoprotein-cholesterol (rho 0.1, p < 0.05). LPA was correlated with maximal aerobic power in both sexes with rho 0.2 for total LPA and 0.4 for vigorous LPA (p < 0.01). In men, similar correlations were found for changes in total vigorous PA.

The overall energy expenditure reported was 18% higher in summer than in winter. The amount of total and commuting PA in the two seasons were highly correlated with rho values of 0.9 and 0.7, respectively (p < 0.01).

**Conclusion:**

Weak, but consistent correlations with baseline biological and anthropometrical measurements were found in both sexes, but for changes in PA such a pattern was seen in men only. The total energy expenditure in summer and winter were highly correlated although the absolute volume was higher in summer than in winter.

## Background

The International Physical Activity Questionnaire (IPAQ) was developed in the late 1990s to obtain internationally comparable data on health-related physical activity (PA) [1,2]. Reliability and validity results for the first versions of IPAQ were reported in 2003, showing correlations (rho) with motion detectors of 0.30–0.33 [2]. Later, a revised IPAQ-L version has been launched [1]. Both versions measure vigorous and moderate PA at work, for transportation, on the domestic and the leisure-time arena and time spent sitting on a weekday and on a weekend day. As different from the first, the revised version does not aim to measure low-intensity PA. The first version had alternative forms assessing PA in "a usual week" and "the last 7 days", respectively, but after the revision the latter form is recommended.

The assessment of criterion validity for a PA questionnaire implies the use of a direct method, such as measurement by motion detector, doubly labelled water technique or direct observation of PA [3,4]. Validation by use of a direct method is needed to estimate the absolute amount of PA and most relevant when monitoring adherence to health enhancing PA recommendations. Most often, only modest associations of self-reports with accelerometers and other direct PA measures are reported, as with the first IPAQ version [2]. However, for cohort studies relating PA to health outcomes, questionnaires are expected to rank the study population by categories of habitual PA over a longer time period. For this purpose precise absolute estimates of PA are not critically important, and a questionnaire's consistency with variables known to be related to PA, such as body mass index (BMI), indicators of lipid and glucose metabolism, maximal aerobic power and muscular strength are relevant validation characteristics [4]. Such correlations have been referred to as indirect or construct validity [4,5].

The Romsås in Motion Study was a quasi-experimental population-based community study set up in 2000 to promote PA and evaluate a theory-based, multi-component and multilevel intervention in low-income, multiethnic districts of Oslo with high mortality rates [6,7]. Based on the promising preliminary report from the validation of the IPAQ [8], we decided to include IPAQ-L as well as two short leisure time PA (LPA) questionnaires measuring habitual PA and previously used in health surveys in Norway [6]. As outdoor PA is heavily influenced by weather and season, we used the self-administered IPAQ-L in the version assessing PA in a usual week [2] adapted to Nordic countries with separate questions for summer and winter [6]. The aim of this paper is to present the associations of PA expenditure measured by this version of IPAQ-L with anthropometrical and biological measurements [6] and aerobic power [9] and to report the seasonal variations in PA measured with IPAQ-L.

## Materials and methods

The study is based on data from the Romsås in Motion community based intervention study. The total population 31–67 years of age in the intervention district (Romsås) and an age-matched sample from a control district with similar population characteristics were invited (N = 6140). The questionnaires, measurements and main results have been described earlier [6,7]. The main survey questionnaire (Q1), filled in at home, prior to the attendance, provided information on general health, specific diseases and smoking habits, short questions on LPA and education attainment. A supplementary form, handed out by the survey team (Q2), contained an adapted version of IPAQ-L and questions on psychosocial variables related to PA. Q2 could be filled in on site or returned by prepaid mail. Both questionnaires were available in Norwegian, English, Urdu, Turkish, Vietnamese and Tamil. Three sport science students encouraged the filling-in and noted problems that were presented, not, however, by systematic interviews.

Based on demographic and socio-economic variables, the baseline participants (N = 2950, 48%) were fairly representative of the invited population [6]. A total of 2274 persons (77%) completed IPAQ-L in Q2, and 2240 subjects (men: 1068, women: 1372) had data on relevant survey measurements and were included in the analyses. Baseline participants still alive and living in the Oslo area in 2003 (N = 2644) were invited to the follow-up survey in 2003, 1766 (67%) attended [7], and 1271 had complete IPAQ-L forms at both surveys (conducted during the spring, from March to May). In the intervention district, participants were invited to fitness tests shortly after both surveys [9], and 162 men and 231 women with complete survey data took part in this test at baseline.

The Regional Ethics Committee and the Norwegian Data Inspectorate approved the study protocol. The participants included in analysis have given written consent for the use of their data.

### Anthropometrical and biological measurements

Body weight (in kg, one decimal) and height (in cm, one decimal) were measured in light clothes with an electronic device (DS 102, Arctic Heading, Norway) [6]. BMI was calculated as kg/m^2^. The waist and hip circumferences were measured with light clothes in standing position with a flexible steel device, the waist at the maximum measure around umbilicus and the hip at its maximum. Waist-to-hip ratio was computed as waist circumference/hip circumference.

Resting blood pressure and heart rate were measured with Dinamap, model 8100/8101, Criticon, Tampa, USA, according to established standards [6]. Non-fasting blood samples were analysed for serum total cholesterol (TC), high-density lipoprotein (HDL)-cholesterol, triglycerides (TG) and glucose.

Aerobic power was estimated by a walk test, with two kilometre of fast walking at constant speed and measurement of heart rate at the finish line (the UKK walk test) [9]. Maximal aerobic power (maximal oxygen consumption in millilitre per kg body weight per minute) and a fitness index, giving aerobic power as a percentage of the predicted sex-specific age mean, were calculated based on time spent and heart rate [9].

### The Romsås study version of IPAQ-L

We used the original IPAQ-L, usual week form, adapted to Nordic seasonal variation. This version included the assessment of walking and biking at low intensity, and had 31 questions in contrast to the 27 questions of the revised version [1]. The subjects were asked to recall the number of days, hours and minutes they engaged in PA of different intensities in each PA domain. Bouts of PA of 10 minutes' duration or more were to be reported and the intensity graded as vigorous or moderate. Walking and bicycling were classified as fast, moderate or slow. We asked for PA in summer and winter using double sets for most questions (a total of 59 questions).

IPAQ-L was used to assess energy expenditure in total, by moderate and vigorous intensity, and by each activity domain including time spent sitting [1,2]. Energy expenditure is expressed as metabolic equivalents multiplied with time in minutes per week (METs*minutes*week^-1^, abbreviated METs-min) [9]. One MET is defined as 3.5 ml O_2 _× kg^-1 ^× min^-1^. Sitting is set to 1 equivalent, slow walking to 2.2, moderately fast walking to 3.3, fast walking to 5, slow cycling to 4, moderate fast cycling to 6, fast cycling to 8, general moderate PA to 4 and general vigorous PA to 8 metabolic equivalents.

### Statistical analysis

Low education was defined as 12 years' or shorter of formal education and low income as less than the median. Immigrants born in Western Europe, North America, Australia and New Zealand were categorised as westerners and all other immigrants as non-westerners. For the analyses the numbers vary somewhat according to attendance and valid measurements. METs-min computed from IPAQ-L was used as a continuous variable.

Differences between two groups were identified by chi square, unpaired t-tests or Mann-Withney-tests. The correlations of self-reported PA with anthropometrical and biological measurements were assessed by use of Spearman's rank correlation coefficient (rho). Changes from 2000 to 2003 were correlated by use of individual delta values computed for PA and relevant indicators. Correlations were tested for significance by the Z-test. All p-values are two-tailed. Data analyses were performed with SPSS 12.0 (SPSS, Inc. Chicago, IL, USA).

## Results

Participants completing the IPAQ-L had higher educational and employment attainment, better health in general and higher commitment to vigorous LPA compared to non-adherers (Table 1). Non-western immigrants constituted 24% of the invited population, 22% of the survey participants, and 16% of the IPAQ-L population.

**Table 1:** Characteristics of the Romsås in Motion Study participants by compliance to IPAQ-L

	IPAQ-L complete, N = 2274	IPAQ-L missing or incomplete, N = 676	P-value^2^
Age, mean (SD)	47.1 (9.8)	46.7 (10.1)	NS
Education, years, mean (SD)	12.1 (3.7)	11.1 (4.1)	< 0.001
Full-time employment, %	64	57	< 0.01
"Western" country of birth, %	84	60	< 0.001
Good health in general, %	69	58	< 0.001
No regular hard LPA last year^1^, %	37	47	< 0.001
BMI, kg/m^2^, mean (SD)	26.8 (4.7)	26.8 (4.6)	NS

^1^According to a short questionnaire on hard leisure-time physical activity (LPA) in the main study questionnaire [6]. ^2^Chi-square test or unpaired t-test, p-values are two-tailed.

The METs-min distributions were skewed in all PA domains except time spent sitting, with the mode at zero or next to it, most marked for occupational PA. LPA and commuting PA combined constituted 24% of the METs-min in men and 26% in women. Occupational PA contributed with one third of the METs-min for those employed. Time spent sitting was close to the normal distribution with a median value of 38 hours per week, and significantly more time was spent sitting by persons with high versus low education (p < 0.001). Of the total METs-min computed from IPAQ-L, 27% referred to activities when sitting.

### Correlations of the adapted IPAQ-L with biological measurements

For both men and women the strongest correlations between energy expenditure according to IPAQ-L and relevant measurements were found for vigorous LPA and for vigorous PA within all domains (Table 2). The correlations were statistically significant for all measures in the predicted direction, except for BMI and total vigorous PA in men. For vigorous LPA rho values of -0.14 with waist/hip ratio (men) and -0.17 with TG (women) were reached. Total LPA and total PA within all domains were significantly correlated with some of the measurements only, and the rho values were slightly lower. In men, correlations with occupational PA were similar to those with total PA.

**Table 2:** Correlations of METs-min measured by IPAQ-L with measurements. Rho values.

*METs-min*	*DBP*	*BMI*	*Waist/hip*	*Triglycerides*	*HDL-c.*	*Glucose*
*Men, N = 1068*						
Total PA, all domains	-.08**	-.02	-.12***	-.03	.03	-.00
Commuting PA	-.02	-.01	-.02	.01	.03	.04
Occupational PA	-.08**	-.03	-.09**	-.01	-.02	-.07*
Domestic PA	-.02	.01	-.03	.01	-.00	.03
LPA	-.04	.01	-.09**	-.08**	.10**	-.03
Hours spent sitting	-.02	.06	.04	-.01	.00	-.03
*PA by intensity*						
Vigorous LPA	-.13***	-.07*	-.14***	-.13***	.12***	-.07*
Vigorous PA	-.11***	-.04	-.12***	-.07*	.08**	-.07*
Moderate PA	-.05	-.01	-.07*	.03	-.02	.02

*Women, N = 1372*						
Total PA, all domains	-.07*	-.04	-.09**	-.07**	.03	-.09**
Commuting PA	-.05	-.05	-.07*	-.04	.04	-.02
Occupational PA	-.03	.02	-.02	-.03	-.00	-.03
Domestic PA	-.03	-.02	-.01	-.02	.02	-.04
LPA	-.05	-.10**	-.13***	-.14***	.09**	-.10***
Hours spent sitting	.06*	.01	-.01	-.05	-.01	-.03
*PA by intensity*						
Vigorous LPA	-.08**	-.09**	-.12***	-.17***	.07*	-.12***
Vigorous PA	-.07**	-.06*	-.12***	-.13***	.08**	-.10***
Moderate PA	-.06*	-.02	-.05	-.03	-.00	-.07*

p-values according to Z-test: * p < 0.05; ** p < 0.01; *** p < 0.001.

The rho values were slightly higher in women than in men for BMI, TG and glucose, but the reverse was seen for HDL-cholesterol and DBP (Table 2). In women total moderate PA and moderate LPA correlated negatively with SBP (rho -0.1, p < 0.05, not shown in table), and moderate LPA showed a similar correlation with serum TC in both sexes.

In the sub-sample conducting the walk test, 51% of the men and 65% of the women reported no vigorous LPA. The correlations of PA measured by IPAQ-L with maximal aerobic power were 0.2 for total LPA and 0.3–0.4 for vigorous LPA (p < 0.01).

Correlations between changes in PA and changes in biological measurements from 2000 to 2003 are shown in Table 3. In men, changes in vigorous PA within all domains were negatively correlated to changes in body weight (rho = -0.12) and positively with changes in HDL-cholesterol (rho = 0.12). Change in vigorous LPA was positively correlated to change in VO_2 _max (rho = 0.31), and change in total PA and occupational PA were correlated positively to change in HDL-cholesterol. In women, no correlations for changes were found in the total sample, whereas in the subgroup attending the walk tests, changes in total LPA was positively correlated to changes in the fitness index (rho = 0.32).

**Table 3:** Correlation of changes (delta values) in METs-min according to IPAQ-L with changes in measurements 2000–2003. Rho values.

*METs-min*	*Weight*	*Triglycerides*	*HDL-chol.*	*Fitness index*	*VO*_2_*max*
*Delta values*	*Men, N = 559*			*N = 45*	
Total PA	-.06	-.01	.10*	.19	.20
Occupational PA	-.05	-.05	.16***	-.06	-.05
LPA	-.07	.10*	.01	.15	.17
Hours spent sitting	.05	-.02	-.07	.25	.23
*PA by intensity*					
Vigorous LPA	-.01*	.04	-.01	.28	.31*
Moderate LPA	.05	-.09*	-.01	.11	.11
Vigorous PA	-.12**	-.01	.12**	.21	.21
Moderate PA	-.03	.00	.06	.25	.26

	*Women, N = 712*			*N = 55*	
Total PA	-.04	-.01	.039	.03	.12
Occupational PA	-.02	-.01	.03	-.05	.10
Domestic PA	.01	-.01	-.03	.16	.18
LPA	-.02	-.01	-.04	.32*	.19
Hours spent sitting	.08	-.07	.023	.16	.09
*PA by intensity*					
Vigorous LPA	-.02	-.07	.047	.19	.09
Moderate LPA	.02	.03	.053	-.25	-.14
Vigorous PA	-.02	-.05	.052	.03	.10
Moderate PA	-.03	.01	.024	.03	.10

p-values according to Z-test: * p < 0.05; ** p < 0.01; *** p < 0.001.

### Seasonal and weekly variation in PA

The total PA energy expenditure was 18% higher in summer than in winter (p < 0.01) and was higher in summer in all domains except occupational PA. More time was spent sitting in winter than in summer, by 8% in men and 10 % in women (p < 0.01). The amount of total and commuting PA in the two seasons were highly correlated with rho values of 0.91 and 0.68, respectively (p < 0.01). The total number of METs-min by walks in weekends was higher by 43% (p < 0.01) compared with working days. LPA in working days and weekends was highly correlated (rho = 0.54, p < 0.01), as was time spent sitting (rho 0.61 (men), 0.67 (women), p < 0.01).

### Absolute volumes of PA

The absolute total PA volumes did not differ significantly by education for women. Men with low education reported higher total energy expenditure than men with high education due to more moderate PA (table 4). Subjects with high education reported the highest volumes of LPA for both sexes, and for women also of vigorous PA. The median total energy expenditure in men of non-western origin was 5158 (25–75 percentiles 1684–13870) METs-min, compared with 4248 (2027–9333) in western men. Non-western women reported PA of 5519 (1665–15461) METs-min and western women 4232 (2282–8498) METs-min. Non-western immigrants of both sexes reported less energy spent on LPA and less time sitting, but more energy spent at work compared with westerners.

**Table 4:** Absolute energy expenditure in METs-min according to IPAQ-L at baseline. Median values by sex and education.

	Education ≥ 12 years		Education < 12 years	
*Men, N = 1068*	*N*	*METs-min*	*N*	*METs-min*
Total PA	577	3976	399	5670*
Commuting PA	615	363	430	366
Occupational PA	606	289	425	440
Domestic PA	615	420	431	495
Hours spent sitting	616	44	435	35***
LPA	587	1230	431	891***
*PA by intensity*				
Vigorous PA	609	960	425	720
Moderate PA	583	2520	404	3323**

*Women, N = 1372*				
Total PA	624	4533	623	4197
Commuting PA	656	471	674	406
Occupational PA	656	53	678	0
Domestic PA	658	757	680	945
LPA	634	1389	632	1024***
Hours spent sitting	661	40	683	32***
*PA by intensity*				
Vigorous PA	656	480	677	0***
Moderate PA	626	3450	626	3561

p-values according to Mann-Whitney tests: * p < 0.05; ** p < 0.01; *** p < 0.001.

## Discussion

Total vigorous PA, vigorous LPA and total PA within all domains recorded by the Romsås in Motion Study version of IPAQ-L were consistently correlated with anthropometric and biological measurements in the baseline survey. The total energy expenditure was 18% higher in summer than in winter, and the correlation of total PA in the two seasons was high.

The correlation coefficients for IPAQ-L with relevant survey measurements were low with maximum rho values of -0.17. The most consistent and strongest correlations were found for vigorous PA, while total PA and moderate PA showed less consistent and weaker correlations. Domestic PA, sitting, commuting PA in men and occupational PA in women did not show any correlation of interest with relevant measurements.

A recent validity study in Sweden used the revised IPAQ-L version among 46 healthy volunteers and found correlations with rho values 0.25 for total PA with BMI and 0.21 for total PA with aerobic fitness measured by a sub-maximal treadmill walking test [5]. The lower correlations in our cohort may mainly be due to differences between the populations studied. Our study was done in low-income districts, the mean BMI values were nearly 27 kg/m^2^, and prevalence of obesity (BMI > 30 kg/m^2^) was 37% in non-western women and around 20% in other subgroups [10]. In the Swedish study the educational level was above the national average and the mean BMI was 24 kg/m^2 ^[5]. In our subgroup of volunteers for the fitness test the correlations between vigorous PA and physical fitness were close to the findings in the Swedish study.

PA is known to be overestimated by any type of self-report. This problem seems to be most pronounced for detailed PA instruments, for less intensive PA, and in populations with low absolute PA levels [2,3,11-15]. The 12-country study of IPAQ found a median value of 3699 METs-min in populations described as highly active [2]. In our study the median levels of PA exceeded 4000 METs-min and were even higher in non-western immigrants. Our extra questions to account for seasonal variation, and also the intervention with overweight persons being encouraged to exercise, may have accentuated an over-reporting. The low correlation of changes in PA with changes in relevant measurements among women is striking. A study found that women with hypercholesterolemia were more likely than men to under-report their dietary intake for reasons of social desirability [11]. PA may, similarly, be particularly prone to over-reporting among overweight women.

### Other considerations

Baseline correlations of IPAQ-L and relevant measurements were studied among survey participants for whom all data were available, constituting 36% of all inhabitants in relevant age in the study areas. The correlation of changes from 2000 to 2003 was studied in a subgroup containing 43% of the baseline study population and mixed concerning the intervention, and the correlation of IPAQ-L with physical fitness was assessed in a subgroup of volunteers in the intervention district. These differences are, however, considered not to be a major problem as long as we correlated individual baseline or delta values for PA and for the anthropometrical and biological measures.

We found that the mean waist circumference values differed somewhat between the project nurses, apparently due to variation in measurement method in cases with larger abdominal fat mass [16]. Also, the instruments used for blood pressure measurement showed some variance between them. Such problems make it less likely to detect true associations. The blood samples were taken non-fasting, but postprandial levels of glucose and serum lipids are increasingly recognized as metabolic indicators [17,18].

Nearly all participants started filling-in Q2 at the survey site, but most brought it home after around half an hour's work to finish. The problems most often noted considered the estimation of hours spent sitting and difficulties with referring the PA to the specific domains. Some persons excused themselves for having to mark low PA levels repeatedly and physically impaired persons would have liked to report their reason.

Our total experience supports the decision to abbreviate the IPAQ-L form [1]. If planning the intervention study today, in light of the present knowledge, we had probably chosen the revised IPAQ-L form and aimed at using a motion detector in a sample. Based on our findings one could, however, argue for measuring only the LPA in populations like the one we studied. The construct validity was by far higher for LPA than for PA in other domains, linguistic and over-reporting problems are known to increase with the size of the questionnaire, and the potential for increasing PA by intervention is probably higher for LPA than for PA in other domains.

## Conclusion

A self-administered version of IPAQ-L, adapted to Nordic seasonal variations, was examined for construct validity in a low-income, multi-ethnic cohort in Oslo, Norway. Weak, but consistent correlations with baseline biological and anthropometrical measurements were found in both sexes, but for changes in PA such a pattern was seen in men only. The total energy expenditure was higher in summer than in winter, but total PA in both seasons were highly correlated.

## Competing interests

The author(s) declare that they have no competing interests.

## Authors' contributions

SGI drafted the manuscript. SAA, IH, AKJ and TR participated in the data collection, reviewed and commented the manuscript. AKJ was the primary investigator and IH the senior statistician of the Romsås Study. TR performed all statistical analyses. All authors read and approved the final manuscript.
